# Pt-Au/MO_x_-CeO_2_ (M = Mn, Fe, Ti) Catalysts for the Co-Oxidation of CO and H_2_ at Room Temperature

**DOI:** 10.3390/molecules22030351

**Published:** 2017-02-27

**Authors:** Xiaowei Hong, Ye Sun, Tianle Zhu, Zhiming Liu

**Affiliations:** 1 School of Space and Environment, Beihang University, Beijing 100191, China; hongxiao86@126.com (X.H.); suny@buaa.edu.cn (Y.S.); 2 State Key Laboratory of Chemical Resource Engineering, Beijing University of Chemical Technology, Beijing 100029, China

**Keywords:** CO, H_2_, Co-oxidation, Pt-Au/MO_x_-CeO_2_, Room temperature

## Abstract

A series of nanostructured Pt-Au/MO_x_-CeO_2_ (M = Mn, Fe, Ti) catalysts were prepared and their catalytic performance for the co-oxidation of carbon monoxide (CO) and hydrogen (H_2_) were evaluated at room temperature. The results showed that MO_x_ promoted the CO oxidation of Pt-Au/CeO_2_, but only the TiO_2_ could enhance co-oxidation of CO and H_2_ over Pt-Au/CeO_2_. Related characterizations were conducted to clarify the promoting effect of MO_x_. Temperature-programmed reduction of hydrogen (H_2_-TPR) and X-ray photoelectron spectroscopy (XPS) results suggested that MO_x_ could improve the charge transfer from Au sites to CeO_2_, resulting in a high concentration of Ce^3+^ and cationic Au species which benefits for the CO oxidation. In-situ diffuse reflectance infrared Fourier transform spectroscopy (In-situ DRIFTS) results indicated that TiO_2_ could facilitate the oxidation of H_2_ over the Pt-Au/TiO_2_-CeO_2_ catalyst.

## 1. Introduction

Both H_2_ and CO co-exist in space capsule. H_2_ mainly originates from the charge–discharge process of battery and CO is released from processes of human metabolism. H_2_ and CO, as the typical inflammable and explosive gaseous contaminants, seriously threaten the safety of human and airtight cabin. In addition, long-term exposure to CO causes adverse effects on human health [1]. Therefore, more and more attention has been paid on the simultaneous removal of CO and H_2_. Catalytic oxidation has been regarded as an effective and green technology to eliminate CO and H_2_ [2].

Noble metal catalysts for the oxidation of H_2_ and CO have been extensively studied recently [3,4,5,6,7,8]. Pt catalysts were considered as the most active catalysts for H_2_ oxidation at room temperature [9]. Previous studies showed that Pt-Ru/C [10] and Pt-Sn/C [11] electrocatalysts exhibited high catalytic performance for the H_2_ oxidation, and metallic Pt species were more active than the oxidized Pt species [12,13]. Au catalysts have been reported to be more active than Pt catalysts for CO oxidation [8,14]. CO could be removed at room temperature over Au/CeO_2_ [15], Au/TiO_2_ [16], Au/MnO_x_ [17], and Au/Fe_2_O_3_ [18] catalysts. Moreover, cationic Au species were more active than the metallic Au species [18,19]. 

In order to remove CO and H_2_ in space capsule, developing novel catalysts with excellent activity for the co-oxidation of CO and H_2_ is desirable. Recently, a few studies related to the co-oxidation of H_2_ and CO have been reported [5,6,20,21,22]. Zhang et al. [21] reported that Pt-Au/CeO_2_ bimetallic catalysts with ordered macro-porous and meso-porous structure showed superior catalytic performance for CO oxidation but not for H_2_ oxidation. Ru-Pt bimetallic core-shell nanoparticle catalyst has been developed by Eichhorn et al. [23], however, it could not afford the simultaneous removal of CO and H_2_. Au-Pd/Fe(OH)x catalyst with separate Au active sites and Pd active sites was excellent for the complete co-oxidization of H_2_ and CO at low temperature [5], but the gas hourly space velocity (GHSV) (20,000 h^−1^) was relatively low. At high GHSV, the oxidation of H_2_ was strongly inhibited by the presence of CO [6]. Therefore, the simultaneous removal of H_2_ and CO at room temperature at high GHSV still remains challenging. CeO_2_ enhanced the oxidation reactions due to its high oxygen storage capacity and redox property [24]. Corma et al. [15] pointed out that nanocrystalline CeO_2_ with Ce^3+^ ions could adsorb and activate O_2_, thus enhancing the catalyst reactivity. Ordered CeO_2_ support with higher surface area could lead to the better dispersion of active sites and also boost oxygen transfer to active platinum species [25]. For the CO and H_2_ oxidation, surface diffusion and spillover enhanced oxidation reaction on Pt/CeO_2_ [26,27,28] and Au/CeO_2_ catalysts [8,29]. Therefore, CeO_2_ nanospheres with meso-structure were promising supports for Au and Pt catalysts. Fe_2_O_3_ [30,31,32], TiO_2_ [33,34] and MnO_2_ [35,36,37,38] were proven to be excellent promoters because of their high oxygen storage capacity and redox property. In addition, preparation methods showed significant effect on the catalytic performance of catalysts [6]. Reduction treatment improved the catalytic activities of Pt catalysts [12] and urea was an excellent precipitant for Au catalysts [39].

According to the above-mentioned understanding, a series of nanostructured Pt-Au/MO_x_-CeO_2_ (M = Mn, Fe, Ti) bimetallic catalysts were prepared by the reduction-deposition precipitation method and their performance for the co-oxidation of CO and H_2_ under the GHSV of 500,000 h^−1^ at room temperature were evaluated. Physical and chemical properties of the Pt-Au/MO_x_-CeO_2_ (M = Mn, Fe, Ti) bimetallic catalysts were characterized. Based on the characterization, the relationship between the structure and the catalytic performance has been elucidated.

## 2. Results and Discussion

### 2.1. Catalytic Activities of the Pt-Au/MO_x_-CeO_2_ Catalysts

**Figure 1:**
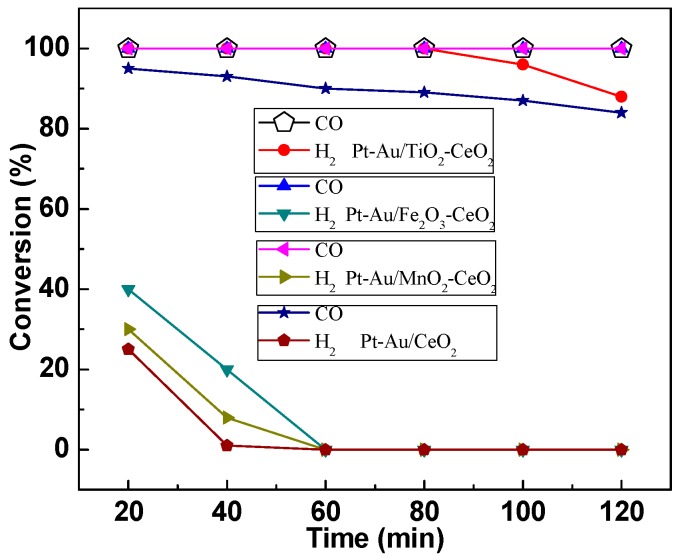
The activities of Pt-Au/MO_x_-CeO_2_ catalysts for the catalytic co-oxidations of H_2_ and CO. Reaction conditions: 100 ppm H_2_/100 ppm CO/room air; temperature: 25 °C; GHSV = 500,000 h^−1^.

Figure 1 presents the activities of the Pt-Au/MO_x_-CeO_2_ catalysts for the catalytic co-oxidation of CO and H_2_. For Pt-Au/CeO_2_ catalyst, conversions of CO and H_2_ are 93% and 25%, respectively, and then gradually decrease. It is attributed to the CO accumulation on Au and Pt active sites. CO can be completely removed while the conversion of H_2_ is low over Pt-Au/MnO_2_-CeO_2_ and Pt-Au/Fe_2_O_3_-CeO_2_ catalysts. It is encouraging that 100% conversions of CO and H_2_ are obtained at room temperature over Pt-Au/TiO_2_-CeO_2_ catalyst. However, the conversion of H_2_ decreases over Pt-Au/TiO_2_-CeO_2_ catalyst due to H_2_O accumulation on the Pt and Au active sites [7]. The oxidation of CO is suppressed by H_2_O when the H_2_O content is over 200 ppm [40]. The inhibiting effect of H_2_O is due to the competitive adsorption between H_2_O and CO molecules on the surface twofold coordinated oxygen site [41]. On the other hand, a competitive adsorption between H_2_O and O_2_ molecules also exists due to the accumulation and occupation of H_2_O on the Pt and Au active sites [7].

### 2.2. Physicochemical Properties of Catalysts

**Figure 2:**
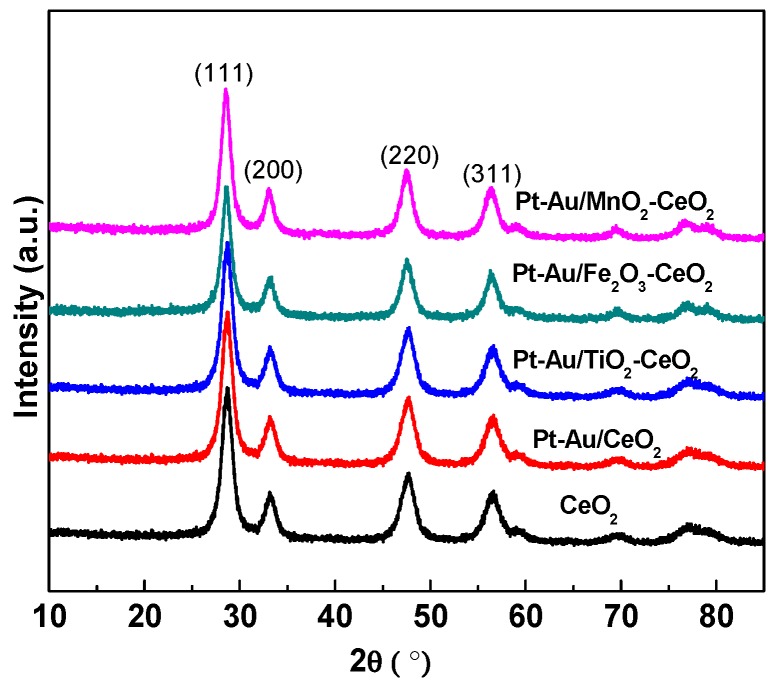
XRD patterns of CeO_2_ support and Pt-Au/MO_x_-CeO_2_ catalysts.

**Figure 3:**
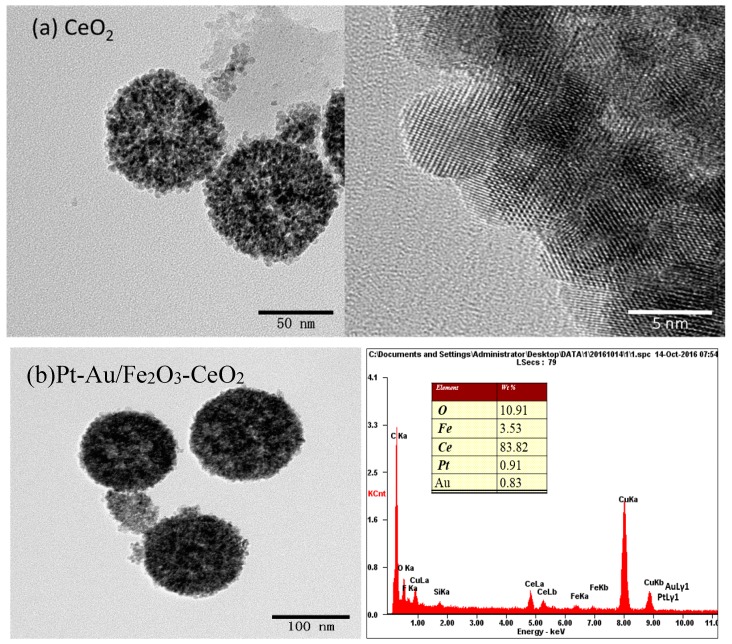

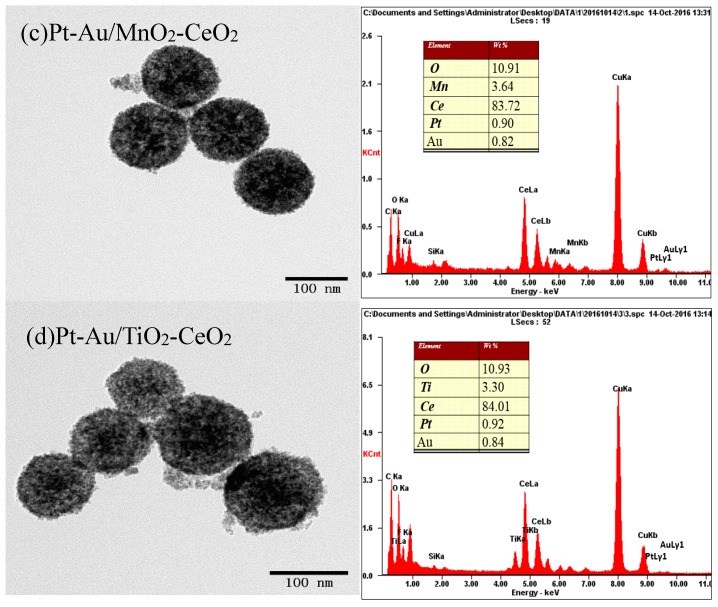
TEM image of the CeO_2_ support and EDS results of Pt-Au/MO_x_-CeO_2_ catalysts: (**a**) Pt-Au/CeO_2_; (**b**) Pt-Au/Fe_2_O_3_-CeO_2_; (**c**) Pt-Au/MnO_2_-CeO_2_; (**d**) Pt-Au/TiO_2_-CeO_2_.

Figure 2 presents the X-ray diffraction (XRD) patterns of the CeO_2_ support and Pt-Au/MO_x_-CeO_2_ catalysts. All these samples show typical cubic CeO_2_ diffraction peaks (JCPDS 43-1002). The diffraction peaks ascribed to MO_x_, Pt and Au species are absent, which indicates that MO_x_, Pt and Au species are highly dispersed on the support. Figure 3 shows the transmission electron microscope (TEM) images of the CeO_2_ support and Pt-Au/MO_x_-CeO_2_ catalysts. It can be found that CeO_2_ support presents nanosphere that is comprised of many small particles with a crystallite size of 5 nm. The contents of Pt and Au species in energy dispersive spectrometer (EDS) results of the Pt-Au/MO_x_-CeO_2_ catalysts are close to the theoretical values (1 wt %). Chemical composition and textural properties of Pt-Au/MO_x_-CeO_2_ catalysts are seen in Table 1. Compared with the X-ray photoelectron spectroscopy (XPS) results presented in Table 1, contents of Pt and Au species in EDS results are higher, indicating that parts of Pt and Au species are distributed on the surface of the CeO_2_ nanoparticles. Brunauer–Emmett–Teller (BET) surface areas of Pt-Au/MO_x_-CeO_2_ catalysts decrease due to the introduction of MO_x_. The dispersions of metal on Pt-Au/MO_x_-CeO_2_ catalysts are very close due to the same preparation method.

**Figure 4:**
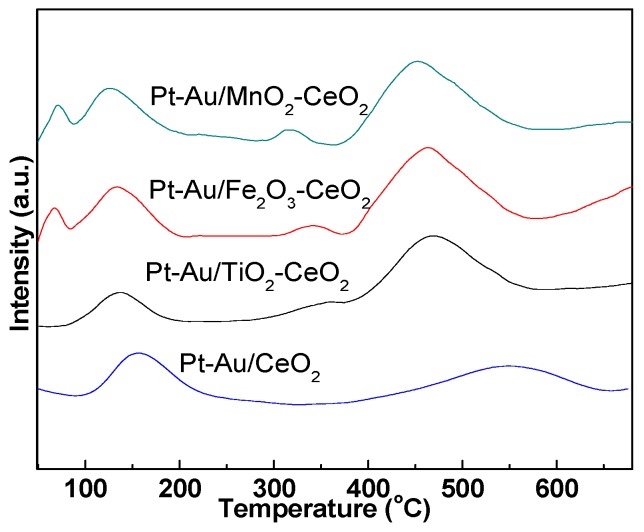
H_2_-TPR profiles of Pt-Au/MO_x_-CeO_2_ catalysts.

Figure 4 shows the H_2_-TPR profiles of Pt-Au/MO_x_-CeO_2_ catalysts. The reduction temperatures of Pt species are 70–100 °C [42]; Au species reduction temperatures are usually 100–200 °C [43]; and pure CeO_2_ reduction temperature is around 553 °C [44]. The peak at 350 °C is attributed to the reduction of CeO_2_ surface oxygen [45]. Evidently, no reduction peaks ascribed to Pt species are observed for Pt-Au/CeO_2_ and Pt-Au/TiO_2_-CeO_2_ catalysts, suggesting that all the Pt species are metallic Pt species. For Pt-Au/MnO_2_-CeO_2_ and Pt-Au/Fe_2_O_3_-CeO_2_ catalysts, the reduction peak at 75 °C is attributed to Pt^2+^ species. For Pt-Au/CeO_2_ catalyst, there are two reduction peaks at 160 and 553 °C, which are ascribed to the reduction peaks of Au species and CeO_2_, respectively [46]. Three reduction peaks at 146, 350 and 465 °C are observed for Pt-Au/TiO_2_-CeO_2_ catalyst. It can be observed that three reduction peaks are at 144, 341 and 450 °C for Pt-Au/MnO_2_-CeO_2_ catalyst. The reduction peaks are centered at 145, 350 and 464 °C for Pt-Au/Fe_2_O_3_-CeO_2_ catalyst. It is worth noting that the reduction temperature of CeO_2_ in Pt-Au/MO_x_-CeO_2_ is lower than that of Pt-Au/CeO_2_ nearly by 100 °C, which means that the oxidative performance of CeO_2_ in Pt-Au/MO_x_-CeO_2_ is higher than that of Pt-Au/CeO_2_. On the other hand, the introduction of MO_x_ influences the reduction temperature of Au species. Lower reduction temperature of Au species indicates the active oxygen species formed on the Pt-Au/MO_x_-CeO_2_ catalysts are more active [43]. It is very interesting that, even though the Pt species should be metallic Pt due to the reduction of NaBH_4_ over the Pt-Au/MnO_2_-CeO_2_ and Pt-Au/Fe_2_O_3_-CeO_2_ catalysts, Pt^2+^ species is observed. The presence of Pt^2+^ species can be caused by the addition of MnO_2_ and Fe_2_O_3_, both of which improve the electron transfer from Pt sites to CeO_2_, thus leading to the oxidation of metallic Pt species to Pt^2+^ species. Metallic Pt species are more active than Pt^2+^ species for the oxidation reaction, which may clarify the poor activities of Pt-Au/MnO_2_-CeO_2_ catalyst and Pt-Au/Fe_3_O_4_-CeO_2_ catalyst for the H_2_ oxidation. H_2_ consumed amounts are 0.21, 0.31, 0.38 and 0.39 mmol^−1^ for the Pt-Au/CeO_2_, Pt-Au/TiO_2_-CeO_2_, Pt-Au/Fe_2_O_3_-CeO_2_, and Pt-Au/MnO_2_-CeO_2_ catalysts, respectively. It indicates that the addition of MO_x_ can promote the redox property of Pt-Au/CeO_2_ catalyst.

**Figure 5:**
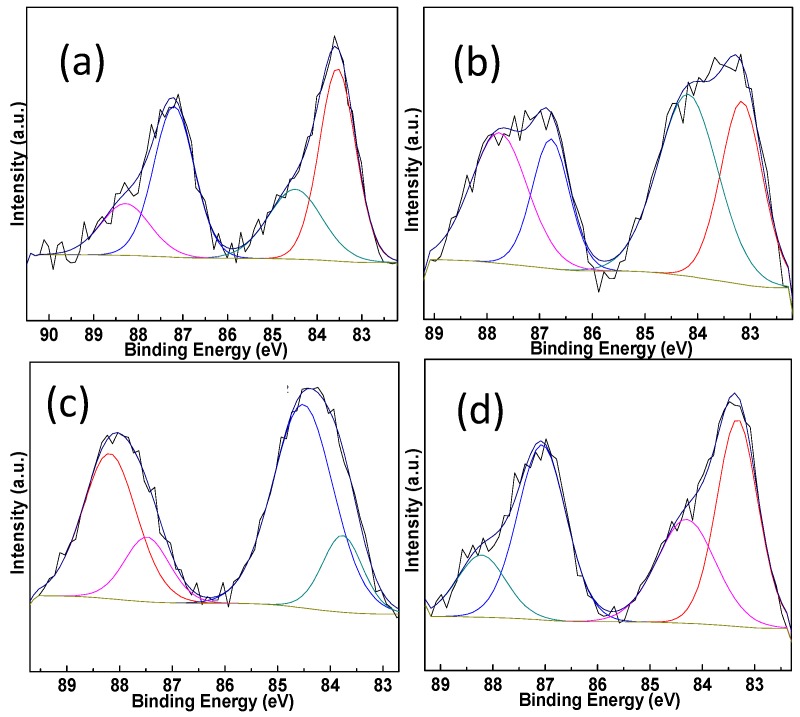
Au 4f XPS spectra of Pt-Au/MO_x_-CeO_2_ catalysts: (**a**) Pt-Au/CeO_2_; (**b**) Pt-Au/Fe_2_O_3_-CeO_2_; (**c**) Pt-Au/MnO_2_-CeO_2_; (**d**) Pt-Au/TiO_2_-CeO_2_.

**Figure 6:**
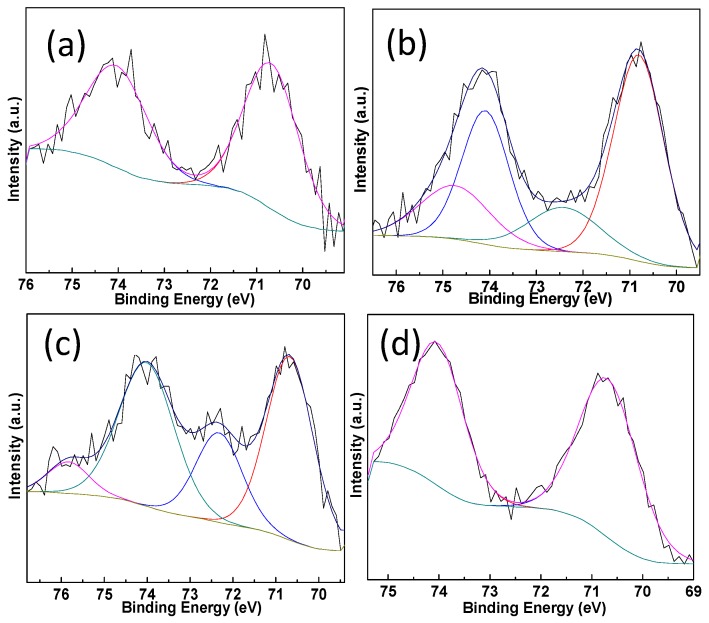
Pt 4f XPS spectra of Pt-Au/MO_x_-CeO_2_ catalysts: (**a**) Pt-Au/CeO_2_; (**b**) Pt-Au/Fe_2_O_3_-CeO_2_; (**c**) Pt-Au/MnO_2_-CeO_2_; (**d**) Pt-Au/TiO_2_-CeO_2_.

**Figure 7:**
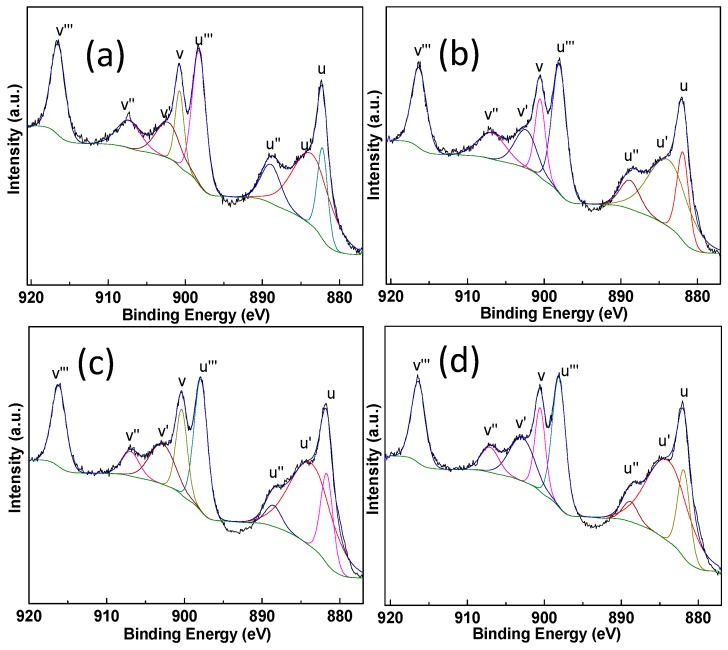
Ce 3d XPS spectra of Pt-Au/MO_x_-CeO_2_ catalysts: (**a**) Pt-Au/CeO_2_; (**b**) Pt-Au/TiO_2_-CeO_2_; (**c**) Pt-Au/Fe_2_O_3_-CeO_2_; (**d**) Pt-Au/MnO_2_-CeO_2_.

**Figure 8:**
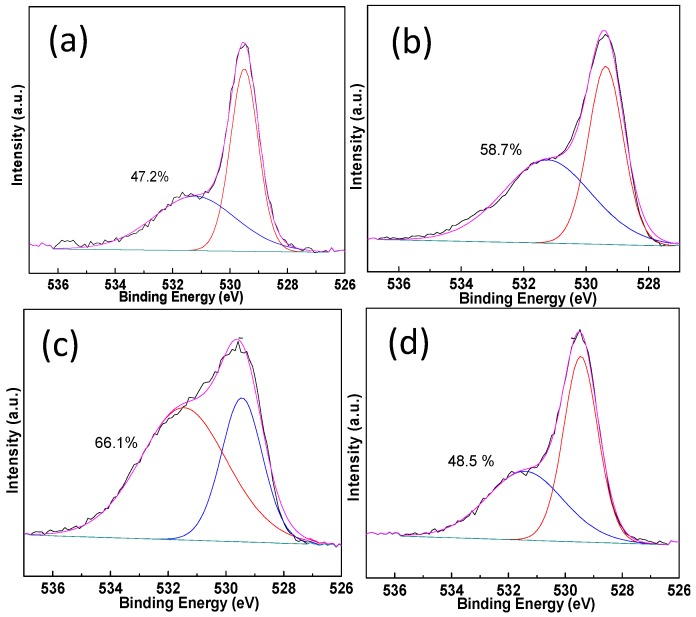
O1s XPS spectra and O_II_ ratios of Pt-Au/MO_x_-CeO_2_ catalysts: (**a**) Pt-Au/CeO_2_; (**b**) Pt-Au/Fe_2_O_3_-CeO_2_; (**c**) Pt-Au/MnO_2_-CeO_2_; (**d**) Pt-Au/TiO_2_-CeO_2_.

XPS measurements are conducted on the Pt-Au/MO_x_-CeO_2_ samples and the results are listed in Table 2. The peaks at 83.3–83.6 eV and 84.2–84.5 eV can be assigned to Au^0^ species and Au^+^ species, respectively [46,47]. The peaks at 70.2–70.8 eV and 72.4–72.6 eV are attributed to Pt^0^ species and Pt^2+^ species [48]. Figure 5 and Figure 6 show that the addition of MO_x_ influences the chemical states of Pt species and Au species due to the electron transfer from Au species and Pt species to CeO_2_ [49,50,51,52]. Cationic Au species possess higher activity than metallic Au species on the CO oxidation [42]. Compared with Pt-Au/CeO_2_ catalyst, the addition of MO_x_ results in the presence of more cationic Au species over Pt-Au/MO_x_-CeO_2_ catalysts. The Ce 3d XPS peaks were fitted by searching for the optimum combination of Gaussian bands with the correlation coefficients (r^2^) above 0.99. In Figure 7, the Ce 3d core level spectra of the catalyst can be divided into eight components and the content of Ce^3+^ are listed in Table 2. The bands labeled u′ and v′ represent the 3d^10^4f^1^ corresponding to Ce^3+^, and the bands labeled u, u′′, u′′′, v, v′′, and v′′′ represent the 3d^10^4f^0^ corresponding to Ce^4+^ [53]. Among Pt-Au/MO_x_-CeO_2_ catalysts, the content of Ce^3+^ over Pt-Au/MnO_2_-CeO_2_ catalyst is the highest, indicating that more surface oxygen vacancies exist on Pt-Au/MnO_2_-CeO_2_ catalyst. Previous researches showed that the formation of Ce^3+^ over Au/CeO_2_ catalysts was due to the charge transfer between Au sites and CeO_2_ [49,50,51]. Therefore, the introduction of MO_x_ enhances the charge transfer from Au species and Pt species to CeO_2_ and leads to high content of cationic Au species. O1s XPS spectra of Pt-Au/MO_x_-CeO_2_ catalysts are shown in Figure 8 and two peaks at 529.1–529.4 and 531.1–531.4 eV, respectively, appear. The former is ascribed to lattice oxygen (O_I_) and the latter is attributed to chemisorbed oxygen (O_II_) [47]. O_II_ ratios [O_II_/(O_II_ + O_I_)] over Pt-Au/MO_x_-CeO_2_ catalysts are higher than that over Pt-Au/CeO_2_ catalysts due to the presence of higher Ce^3+^ content.

**Figure 9:**
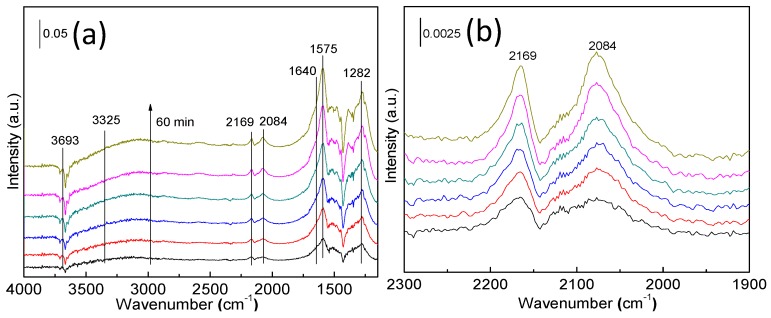

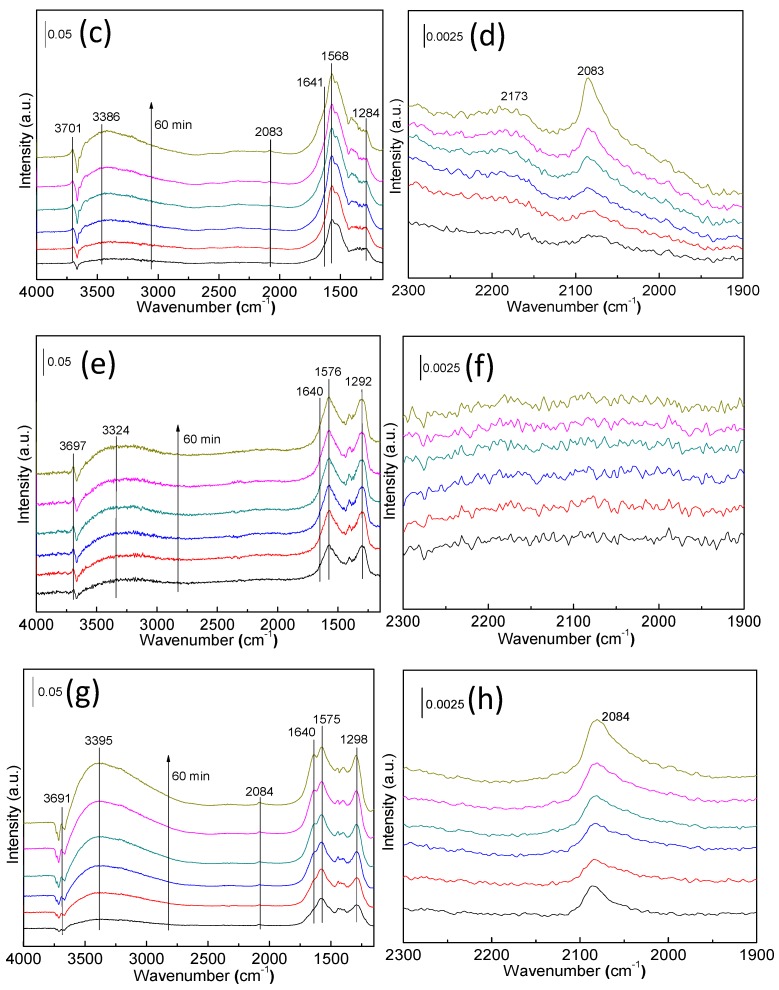
In-situ DRIFT spectra of the Pt-Au/MO_x_-CeO_2_ catalysts after exposed upon 3000 ppm CO + 3000 pm H_2_ + synthetic air for 60 min at 25 °C: (**a**,**b**) Pt-Au/CeO_2_; (**c**,**d**) Pt-Au/Fe_2_O_3_-CeO_2_; (**e**,**f**) Pt-Au/MnO_2_-CeO_2_; (**g**,**h**) Pt-Au/TiO_2_-CeO_2_.

To further understand the relationship between catalyst activity and catalyst physicochemical property, In-situ diffuse reflectance infrared Fourier transform spectroscopy (In-situ DRIFT) spectra of the Pt-Au/MO_x_-CeO_2_ catalysts obtained upon exposure to CO, H_2_ and synthetic air at 25 °C are shown in Figure 9. Seven distinct bands are observed in the in-situ DRIFT spectra. The bands at 3324–3395, 1640, 3691–3701, 2169, 2083–2084, 1568–1575, and 1282–1298 cm^−1^ are ascribed to isolated hydroxyl groups υ(OH) [29], adsorbed water δ(H-O-H) [43], another hydroxyl groups υ(OH) [54,55,56,57,58], the adsorption of CO on Au sites [59,60,61], the adsorption of CO on Pt sites [42,62,63], the carbonate species [29], and carbonate species [29], respectively. It has been proposed that isolated hydroxyl groups originated from the decomposition of the OOH species, which were generated from the reaction between the associatively adsorbed oxygen and dissociative adsorbed hydrogen, and the isolated hydroxyl groups further react with dissociative adsorbed hydrogen to generate H_2_O [7,29].

**Figure 10:**
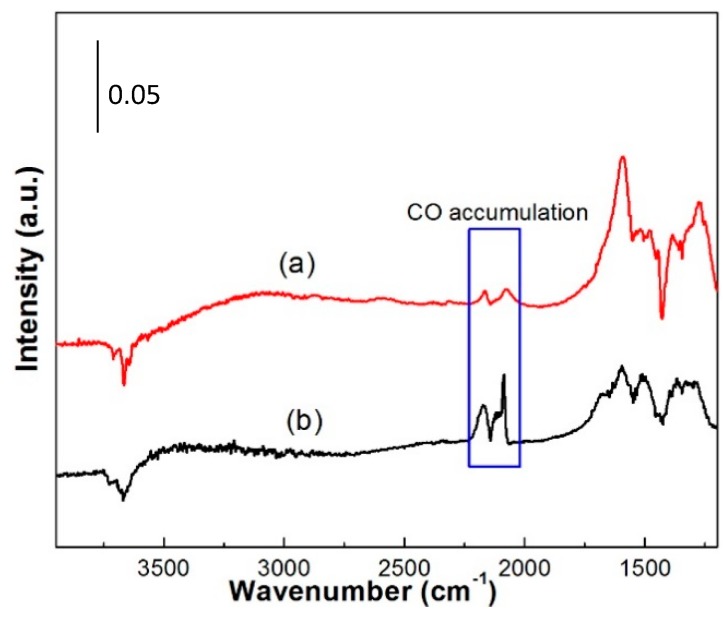
In-situ DRIFTS spectra of the Pt-Au/CeO_2_ catalysts after exposed upon: (**a**) 3000 ppm CO + 3000 ppm H_2_ + synthetic air; and (**b**) 3000 ppm CO + N_2_ for 60 min at 25 °C.

Intensity of reactant-related is due to reactant oxidation and reactant adsorption capability. To solve this problem, In-situ DRIFTS test results of Pt-Au/CeO_2_ catalyst upon 3000 ppm CO + N_2_ and 3000 ppm CO + 3000 pm H_2_ + synthetic air can be observed in the Figure 10. The results indicate that the CO accumulation amount does not reach the CO saturated adsorption capability over Pt-Au/CeO_2_ catalyst upon 3000 ppm CO + 3000 pm H_2_ + synthetic air. CO saturated adsorption capability of Pt-Au/MO_x_-CeO_2_ catalysts may be close to that of Pt-Au/CeO_2_ catalyst due to the close amount of Pt and Au species over Pt-Au/CeO_2_ catalyst and Pt-Au/MO_x_-CeO_2_ catalysts. Little CO accumulation on Pt-Au/MO_x_-CeO_2_ catalysts in Figure 9 is because of the enhanced CO oxidation instead of adsorption capability. For Pt-Au/CeO_2_ catalyst, the intensity of bands at 2169 and 2084 cm^−1^ increases, which means that much CO accumulates on the Au and Pt active sites. The intensity of other bands are almost unchanged, which suggests little H_2_ is oxidized because Pt active sites are occupied and poisoned by CO. Therefore, CO and H_2_ cannot be simultaneously removed over Pt-Au/CeO_2_ catalyst. For Pt-Au/MnO_2_-CeO_2_ catalyst, no peaks can be observed at 2169 and 2084 cm^−1^ with the reaction proceeding, which suggests that the introduction of MnO_2_ enhances the oxidation of CO. However, the intensity of bands at 3389 and 1640 cm^−1^ are seldom unchanged, which indicates that little H_2_ is oxidized. Therefore, the introduction of MnO_2_ improves the oxidation of CO but not the oxidation of H_2_. Over Pt-Au/Fe_2_O_3_-CeO_2_ catalyst, the intensity of the peak at 2084 cm^−1^ suggests that little CO accumulates and the Fe_2_O_3_ improves the activity of Pt-Au/CeO_2_ catalyst for CO oxidation. The intensity of bands at 3386 and 1640 cm^−1^ suggest that little H_2_ is oxidized to H_2_O. Consequently, Fe_2_O_3_ improves the activity of Pt-Au/CeO_2_ catalyst for the CO oxidation not for H_2_ oxidation. Some CO accumulates on the Pt-Au/TiO_2_-CeO_2_ catalyst, as confirmed by the presence of the band at 2084 cm^−1^. The intensity change of peaks at 3691, 3395 and 1640 cm^−1^ suggests that many isolated -OH species and H_2_O are produced, indicating that much H_2_ is oxidized into H_2_O. Based on the in-situ DRIFTS results of the Pt-Au/MO_x_-CeO_2_ catalysts, Pt-Au/TiO_2_-CeO_2_ catalyst presents the best catalytic activity for the co-oxidation of CO and H_2_. 

**Figure 11:**
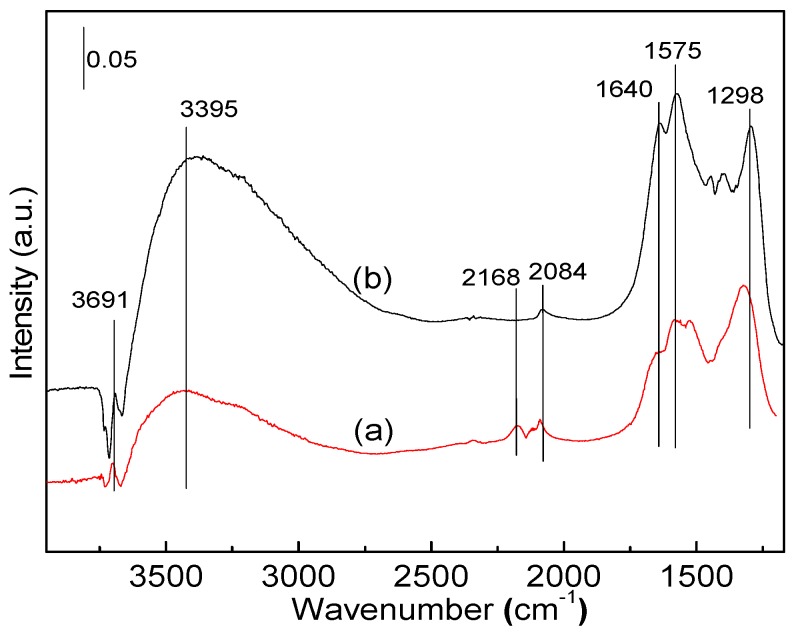
In-situ DRIFTS spectra over Pt-Au/TiO_2_-CeO_2_ catalysts after exposed upon: (**a**) 5000 ppm CO + 3000 ppm H_2_ + synthetic air; and (**b**) 3000 ppm CO + 3000 pm H_2_ + synthetic air for 60 min at 25 °C.

Figure 11 shows the effect of CO concentration on the co-oxidation of CO and H_2_ through in-situ DRIFTS. The intensity of the bands at 3395 and 1640 cm^−1^ in Figure 11b are stronger than that shown in Figure 11a, indicating that more H_2_O are produced. Therefore, CO obviously hinders the oxidation of H_2_ in the co-oxidation of CO and H_2_, which is in accordance with previous reports [22].

## 3. Materials and Methods

### 3.1. Catalyst Preparation

The CeO_2_ nanospheres were prepared by hydrothermal method. Thirteen grams Ce(NO_3_)_3_·6H_2_O was dissolved in 13 mL ultra-pure water at room temperature. Then, 13 mL propionic acid and 390 mL ethylene glycol were added under stirring to form a uniform solution at room temperature. The uniform solution was transferred to a Teflon-sealed autoclave and heated at 180 °C for 7.5 h. After the hydrothermal treatment, the mixture was centrifuged and washed with ethanol for several times. The obtained solid was dried at 100 °C overnight, subsequently it was calcined in air at 400 °C for 4 h. Then CeO_2_ nanospheres were obtained. 

MO_x_-CeO_2_ (M = Mn, Fe, Ti) supports, with a Ce/M molar ratio of 9, were obtained by precipitation method. CeO_2_ nanospheres were homogeneously dispersed in Mn(NO_3_)_2_ or Fe(NO_3_)_3_ aqueous solution, or *tetra*-*n*-butyl titanate ethanol solution, and the suspension was stirred for 2 h at room temperature. Then ammonia solution was added to the above solution under stirring until pH was 10 at room temperature. The suspension was filtered and washed with ultra-pure water. The obtained solid samples were first dried at 105 °C for 12 h and subsequently calcined in air at 400 °C for 4 h to obtain MO_x_-CeO_2_ (M = Mn, Fe, Ti) supports.

Pt-Au/MO_x_-CeO_2_ (M = Mn, Fe, Ti) catalysts were prepared by reduction-deposition precipitation method [49]. Four grams MO_x_-CeO_2_ was uniformly dispersed into the H_2_PtCl_6_ solution containing 0.04 g Pt at room temperature. After impregnation for 2 h, the pH value of suspension was adjusted to 10. NaBH_4_ solution was quickly added into the suspension (NaBH_4_/Pt = 10, molar ratio) while being stirred for 2 h at room temperature. The suspension was filtered and washed with ultra-pure water, then dried under vacuum at 120 °C for 12 h to obtain Pt/MO_x_-CeO_2_. Then 2 g Pt/MO_x_-CeO_2_ was uniformly dispersed into the HAuCl_4_ solution containing 0.02 g Au at room temperature. Subsequently, urea was added as the precipitant. The mixture was stirred at 80 °C for 8 h and then aged for 12 h at room temperature. Then, the mixture was filtered and washed with ultra-pure water. The resulting powder was dried under vacuum at room temperature for 12 h to yield Pt-Au/MO_x_-CeO_2_ catalysts.

### 3.2. Catalyst Characterization

XRD patterns were recorded with a Shimadzu (Tokyo, Japan) XRD-6000 diffractometer operated at 40 kV and 40 mA, using nickel-filtered Cu Kα (λ = 0.1542 nm) radiation. Surface areas of the catalysts were determined by the BET method by a Micromeritics ASAP 2000 instrument (Quantachrome, Boynton Beach, FL, USA). CO chemisorption measurements were measured an Autochem II 2920 (Micromeritics Instrument Corp, Atlanta, GA, USA) automated chemisorption analyzer. The surface chemical states of Pt-Au/MO_x_-CeO_2_ catalysts were tested by XPS (PHI Quantro SXM ULVAC-PHI, Tokyo, Japan) using an Al Kα X-ray source (1486.7 eV) at 15 kV and 25 W with the binding energy calibrated by C 1s at 284.8 eV. HRTEM micrographs were obtained with a JEM-2100F (Jeol, Tokyo, Japan) microscope at 200 kV.

H_2_-TPR measurements equipped with a quadrupole mass spectrometer (Omnistar, Atlanta, GA, USA, GSD-301-O2) were carried out in a fixed bed microreactor. The 0.2-g sample was pretreated under Ar at 120 °C for 1 h. After cooled to 25 °C, the catalyst was reduced under 5% H_2_/N_2_ gas flow (50 mL min^−1^) within the temperature of 30 to 700 °C at 10 °C min^−1^. In-situ DRIFTS were recorded in a Nicolet 6700 FTIR spectrometer (Nicolet, Atlanta, GA, USA). Before characterization, all the catalysts were pretreated under Ar at a flow rate of 100 mL min^−1^ at 120 °C for 0.5 h. After cooled to 25 °C, the reactant gas mixture, comprised of 1000 ppm H_2_, 1000 ppm CO and synthetic air (50% relative humidity), was introduced into the DRIFT cell at a flow rate of 100 mL min^−1^. All spectra were recorded by accumulating 32 scans with a resolution of 4 cm^−1^.

### 3.3. Catalytic Activity Measurement

The activity evaluation for the co-oxidation of CO and H_2_ was performed in a continuous flow fixed-bed quartz reactor (i.d. = 10 mm) by using 0.36 g catalyst at 25 °C. Before activity evaluation, the Pt-Au/MO_x_-CeO_2_ catalysts were pretreated under Ar at 120 °C for 0.5 h at a flow rate of 100 mL·min^−1^. The simultaneous reaction gas consisted of 100 ppm CO and 480 ppm H_2_, and air (50% relative humidity) as the balance gas, and the total flow rate was fixed at 3.6 L·min^−1^, corresponding to a GHSV of 500,000 h^−1^. H_2_, CO and CO_2_ were measured by gas chromatograph (GC) equipped with TCD and FID detectors. CO and CO_2_ were converted to CH_4_ by Ni catalytic converter before the measurement.

## 4. Conclusions

A series of nanostructured Pt-Au/MO_x_-CeO_2_ (M = Mn, Fe, Ti) catalysts were prepared and Pt-Au/TiO_2_-CeO_2_ catalyst presented the best catalytic performance for the total co-oxidation of CO and H_2_ at room temperature. The introduction of MO_x_ into CeO_2_ can enhance the charge transfer from Pt and Au sites to CeO_2_, which improves CO oxidation. The introduction of TiO_2_ enhances the decomposition of OOH species into O species and OH species, while the introduction of MnO_2_ and Fe_2_O_3_ cannot. The addition of TiO_2_ mainly accounts for the high activity for the co-oxidation of CO and H_2_ over the Pt-Au/TiO_2_-CeO_2_ catalyst. 

## Figures and Tables

**Table 1:** Chemical composition and textural properties of Pt-Au/MO_x_-CeO_2_ catalysts.

Catalyst	BET Surface Area (m^2^/g)	Dispersionof Noble Metal	Surface Composition (wt %)	
			Pt	Au
Pt-Au/CeO_2_	156.4	56.3%	0.46	0.51
Pt-Au/Fe_2_O_3_-CeO_2_	125.8	55.7%	0.44	0.48
Pt-Au/MnO_2_-CeO_2_	119.7	55.1%	0.47	0.47
Pt-Au/TiO_2_-CeO_2_	131.3	55.7%	0.46	0.52

**Table 2:** XPS data analysis of Pt-Au/MO_x_-CeO_2_ catalysts.

Catalyst	Pt Species	Content (at. %)	Au Species	Content (at. %)	Ce^3+^ Species Content (at. %)
Pt-Au/CeO_2_	Pt^2+^ (72.5 eV)	0	Au^0^ (83.7 eV)	66.6	34.7
	Pt^0^ (70.7 eV)	100	Au^+^ (84.5 eV)	33.4	
Pt-Au/Fe_2_O_3_-CeO_2_	Pt^0^ (70.7 eV)	77.7	Au^0^ (83.6 eV)	44.4	40.9
	Pt^2+^ (72.5 eV)	23.3	Au^+^ (84.5 eV)	55.6	
Pt-Au/MnO_2_-CeO_2_	Pt^0^(70.7 eV)	67.5	Au^0^ (83.6 eV)	18.4	41.1
	Pt^2+^ (72.5 eV)	32.5	Au^+^ (84.5 eV)	81.6	
Pt-Au/TiO_2_-CeO_2_	Pt^2+^ (72.5 eV)	0	Au^0^ (83.4 eV)	59.2	36.7
	Pt^0^ (70.7 eV)	100	Au^+^ (84.5 eV)	40.8	
